# Integrative multi-omics and bioinformatics analysis of the effects of BaiRui YuPingFeng Powder on intestinal health in broilers

**DOI:** 10.3389/fvets.2025.1606531

**Published:** 2025-06-18

**Authors:** Haonan Xu, Fang Zhang, Yan Che, Yu Cui, Qisheng Yao, Yueqin Guan, Hao Chen, Yuying Huang

**Affiliations:** ^1^College of Animal Science, Anhui Science and Technology University, Fengyang, China; ^2^School of Food Engineering, Anhui Science and Technology University, Fengyang, China; ^3^Jiuhua Huayuan Pharmaceutical Co., Ltd., Chuzhou, China; ^4^College of Life and Health Sciences, Anhui Science and Technology University, Fengyang, China; ^5^School of Pharmacy, Anhui University of Chinese Medicine, Hefei, China

**Keywords:** BaiRui YuPingFeng Powder, broilers, intestinal health, 16S rRNA gene sequencing, metabolomics, bioinformatics

## Abstract

**Introduction:**

In recent years, global poultry consumption has increased rapidly, making chicken the most widely consumed meat worldwide by 2019. To increase livestock development, antibiotics are often added to animal feed as growth promoters. But overuse of antibiotics may alter the gut microbiota, make people more resistant to them, and raise the possibility that they will spread antibiotic resistance genes to the human microbiome. Therefore, identifying safe and effective alternatives to antibiotics in livestock production is crucial for maintaining and improving gut microbial balance, ultimately promoting poultry health. The aim of this study was to investigate the mechanisms behind the impacts of BaiRui YuPingFeng Powder (TCYP) on intestinal health in broilers using combined metabolomics, bioinformatics analysis, and 16S rRNA sequencing.

**Methods:**

In a 42-day feeding trial, 300 one-day-old broilers were randomly divided into five groups (six replicates per group; 10 broilers per replicate) fed a basal diet with or without supplements: control (CON), antibiotic (ATB), and TCYP at 500, 1000, and 1500 mg/kg. Growth performance, serum biochemical parameters, intestinal morphology, cecal microbiota composition, and metabolomic profiles were analyzed. Bioinformatics analysis was used to identify potential targets and pathways, followed by qPCR validation of key genes.

**Results:**

Compared with the CON group, TCYP administration dose-dependently reduced the feed-to-gain ratio (F/G) and average daily feed intake (ADFI) while increasing average daily gain (ADG), with the high-dose TCYP showing more pronounced effects (*p* < 0.05). Serum biochemical analysis revealed that TCYP treatment significantly decreased serum levels of total cholesterol (T-CHO), triglycerides (TG), lactate dehydrogenase (LDH), and alanine aminotransferase (ALT) in a dose-dependent manner, while elevating albumin (ALB) content. These beneficial effects were particularly marked in the high-dose TCYP group (*p* < 0.05). Histopathological examination indicated that high-dose TCYP significantly enhanced villus height and the villus-to-crypt ratio (V/C) in the duodenum, jejunum, and ileum compared to the CON group (*p* < 0.05). 16S rRNA sequencing analysis revealed that TCYP treatment significantly modified the *β*-diversity of cecal microbiota (*p* < 0.01). Compared to the CON group, ATB treatment increased the abundance of *Faecalibacterium and Lachnospiraceae_unclassified* but reduced *Ruminococcaceae_unclassified and Firmicutes_unclassified*. Notably, dietary TCYP supplementation maintained gut microbiota profiles similar to the CON group, demonstrating its stabilizing effect on microbial community structure in broilers. Metabolomic analysis identified differential metabolites primarily involved in lipid and lipid-like molecules, organic heterocyclic compounds, and organic acids and derivatives. Spearman correlation analysis revealed significant associations between *Lachnospiraceae_unclassified* and metabolites such as Gly-Leu, fumarate, and phenylpyruvic acid (|r| > 0.5, *p* < 0.05). Bioinformatics analysis suggested that TCYP may improve intestinal health by regulating key targets, including MMP9, TGFB1, and PPARG, as well as the peroxisome proliferator-activated receptor (PPAR) signaling pathway. Quantitative PCR (qPCR) results showed that, compared to the CON group, TCYP dose-dependently significantly upregulated the mRNA expression of *PPARG, PDPK1*, and *Bcl2* in jejunal tissues (*p* < 0.05), while significantly downregulating the expression of *MMP1* and *Bax* (*p* < 0.05).

**Conclusion:**

TCYP enhances growth performance and intestinal health in broilers through multiple mechanisms, including maintaining cecal microbial homeostasis, modulating lipid and amino acid metabolism, with potential involvement of the PPAR signaling pathway based on bioinformatics and gene expression analysis.

## 1 Introduction

The intricate ecosystem known as the gut microbiota is essential for controlling how nutrients are absorbed and digested as well as blocking the entry of harmful bacteria (1, 2). Butyrate, propionate, and acetate are short-chain fatty acids that are produced as metabolic byproducts when the gut bacteria ferments undigested dietary remnants. In addition to providing the host with energy, these metabolites control intestinal pH and prevent the growth of harmful microorganisms. In chicken, dysbiosis of the gut microbiota can result in decreased immunity, poor nutrient absorption, and digestive issues, raising the risk of intestinal illnesses such bacterial and necrotic enteritis (3, 4). In recent years, global poultry consumption has increased rapidly, making chicken the most widely consumed meat worldwide by 2019 (5). To increase livestock development, antibiotics are often added to animal feed as growth promoters. But overuse of antibiotics may alter the gut microbiota, make people more resistant to them, and raise the possibility that they will spread antibiotic resistance genes to the human microbiome (6). Therefore, identifying safe and effective alternatives to antibiotics in livestock production is crucial for maintaining and improving gut microbial balance, ultimately promoting poultry health.

Herbal feed additives have been widely reported to enhance broiler growth performance and improve gut microbiota composition (7, 8). BaiRui YuPingFeng Powder (TCYP) is a compound herbal formulation derived from YuPingFeng Powder, recorded in Shi Yi De Xiao Fang, with the addition of *Thesium chinense* Turcz. As a classic traditional Chinese medicine prescription, YuPingFeng Powder is composed of *Saposhnikovia divaricata* (Turcz.) Schischk., *Astragalus membranaceus* (Fisch.) Bge., and *Atractylodes macrocephala* Koiz, known for its ability to reinforce Qi, strengthen the body’s defenses, and expel pathogenic factors (9). Modern studies in animal husbandry have demonstrated that YuPingFeng polysaccharides significantly improve gastrointestinal health by regulating immune cell activity, promoting cytokine secretion, and enhancing intestinal barrier function (10, 11). *Thesium chinense* Turcz. is cool in nature, with a pungent, slightly bitter, and astringent taste. It is known for its heat-clearing, detoxifying, and kidney-tonifying properties. According to research, *Thesium chinense* extract is known as a “plant antibiotic” since it successfully suppresses the development of a variety of harmful pathogens, including both Gram-positive and Gram-negative bacteria (12). Furthermore, our earlier research has demonstrated that *Thesium chinense* can improve intestinal barrier integrity, control the makeup of the gut microbiota, and reduce inflammatory responses through the EGFR/PI3K/Akt signaling pathway, all of which help mice with antibiotic-induced diarrhea (13). Nevertheless, the effects of TCYP on intestinal health in chickens warrant further exploration and clarification.

This work evaluated TCYP’s effects on growth performance, cecal microbiota composition, and associated metabolites in broilers using 16S rRNA gene sequencing in combination with fecal metabolomic analysis to better understand how TCYP impacts intestinal health in chickens. Following molecular biological validation, possible targets and pathways linked to TCYP-mediated enhancement of gut health were found using weighted gene co-expression network analysis (WGCNA) based on the GEO database. The purpose of this research is to provide a theoretical framework for TCYP’s use in the poultry sector.

## 2 Materials and methods

### 2.1 Experimental materials and preparation

BaiRui YuPingFeng Powder consists of *Thesium chinense* Turcz., *Saposhnikovia divaricata* (Turcz.) Schischk., *Astragalus membranaceus* (Fisch.) Bge., and *Atractylodes macrocephala* Koiz. in a 2:2:1:1 ratio. *Thesium chinense* was provided by Anhui Jiuhua Huayuan Pharmaceutical Co., Ltd., and identified as *Thesium chinense* Turcz. of the Santalaceae family by Anhui Science and Technology University. The additional medicinal plants were verified to satisfy the requirements of the 2020 edition of the Pharmacopoeia of the People’s Republic of China after being acquired from Anhui Xiehecheng Pharmaceutical Co., Ltd. To obtain TCYP powder, the herbs were decocted twice using distilled water at a 1:10 (g:mL) ratio. The filtrate was then lyophilized. The chemical constituents in TCYP extracts were subsequently identified and analyzed using UHPLC-QE-MS. Detailed analytical methods and corresponding results are provided in Supplementary file S1.

### 2.2 Experimental animals and grouping

The Anhui Science and Technology University Animal Ethics Committee gave its approval to the experimental protocol (Approval No. AK2024045). The animals were handled strictly in line with the *Guide for the Care and Use of Agricultural Animals in Research and Teaching*, guaranteeing adherence to accepted experimental and ethical norms.

Three hundred one-day-old broilers with uniform body weight and an equal number of male and female were acquired from Shandong Yisheng Livestock & Poultry Breeding Co., Ltd., a commercial hatchery. Five sets of sixty chickens each, each consisting of six replicates, 10 birds per replication. Under controlled settings, with an average temperature of 26 ± 4°C and a relative humidity of 60 ± 10%, the experiment was carried out for 42 days in the animal testing area of Anhui Science and Technology University. The broilers had unlimited access to food and water during the feeding session.

The control group (CON) received a standard basal diet, whereas the antibiotic group (ATB) was administered antibiotics through both dietary supplementation and water medication (see Table 1 for specific antimicrobial agents and dosage regimens). In accordance with established research protocols, the treatment groups were allocated TCYP-supplemented diets at graded inclusion levels of 500 mg/kg (TCYP-L), 1,000 mg/kg (TCYP-M), and 1,500 mg/kg (TCYP-H) (14). All experimental diets were formulated to meet the nutritional specifications outlined in the Chinese National Standard GB/T 5916–2020, with corn-soybean meal serving as the basal formulation (complete diet composition presented in Table 2).

**Table 1:** Antibiotic types.

Day-old	Types and dosage of antibiotics
2 ~ 4	10% Spectinomycin (10 g mixed with 20 kg water)
	5% Lincomycin (10 g mixed with 20 kg feed)
8 ~ 11	20% Tilmicosin (10 g dissolved in 40 kg water or 10 g mixed with 10 kg feed)
	10% Amoxicillin (10 g dissolved in 20 kg water or 10 g mixed with 10 kg feed)
14 ~ 16	10% Doxycycline (10 g dissolved in 20 kg water or 10 g mixed with 10 kg feed)
	0.5% Diclazuril Solution (10 mL diluted in 15 kg water)
22 ~ 24	2.5% Danofloxacin Mesylate Powder (10 g dissolved in 20 kg water)
30 ~ 32	10% Colistin Sulfate Premix (10 g dissolved in 20 kg water)

**Table 2:** Composition and nutrient levels of the diets (air-dry basis).

Items (%)	Days 1–21	Days 22–42
Ingredients		
Corn	54.23	56.15
Soybean meal	24.57	23.11
Wheat middling and reddog	5.00	5.00
Wheat bran	2.00	2.00
Peanut meal	3.11	3.00
Cottonseed meal	2.55	2.20
Rapeseed meal	1.99	1.54
Soybean oil	2.00	4.40
Blood meal	2.00	0
NaCl	0.30	0.40
Lys	0.45	0.50
Met	0.15	0.10
Arg	0.20	0.05
Premix^a^	1.45	1.55
Total	100.00	100.00
Nutrient levels^b^		
ME/ (MJ/kg)	12.90	13.50
CP	22.20	19.80
Ca	1.03	1.00
TP	0.71	0.65
Lys	1.30	1.10
Met	0.55	0.48
Arg	0.40	0.35

a The premix provided the following per kg of diets: VA 8200 IU, VD₃ 1,100 IU, VE 8 IU, VK 4.3 mg, VB₁ 3.1 mg, VB₂ 5.1 mg, VB₆ 5.6 mg, VB₁₂ 4.2 mg, folic acid 0.57 mg, niacin 37 mg, Cu 13 mg, Fe 65 mg, Mn 140 mg, Zn 80 mg, and I 0.3 mg.

b ME was a calculated value, while the others were measured values.

### 2.3 Sample collection

On day 42, one bird per replicate was randomly selected for sample collection (*n* = 6). Blood samples were obtained from the wing vein and centrifuged at 3,000 rpm for 15 min at 4°C to separate serum. Concurrently, the chickens were dissected to collect 1 cm tissue segments from the mid-portions of duodenum, jejunum, and ileum. Cecal content samples were immediately transferred to sterile pre-chilled tubes and stored at −80°C for subsequent 16S rRNA sequencing and metabolomics analysis.

### 2.4 Growth performance

Each replicate’s body weight of the fasting broilers was calculated before to the experiment. The following metrics were computed: initial body weight (IBW), final body weight (FBW), average daily gain (ADG), average daily feed intake (ADFI), and feed-to-gain ratio (F/G).

### 2.5 Serum biochemical analysis

Blood samples were collected from the wing vein and centrifuged at 3,000 rpm for 15 min at 4°C to obtain serum. Using commercial test kits from the Nanjing Jiancheng Bioengineering Institute, serum biochemical parameters were evaluated. The following parameters were measured: total cholesterol (TC), triglycerides (TG), albumin (ALB), alanine aminotransferase (ALT) activity, high-density lipoprotein cholesterol (HDL-C), lactate dehydrogenase (LDH), and low-density lipoprotein cholesterol (LDL-C).

### 2.6 Intestinal morphological analysis

For intestinal morphology analysis, six chickens were randomly selected from each group. Tissue samples (1 cm) were collected from the mid-portions of the duodenum, jejunum, and ileum. The samples were fixed in 4% paraformaldehyde for 48 h, embedded in paraffin, and sectioned at approximately 5 μm thickness. Three sections were prepared for each intestinal segment. For each section, three non-overlapping fields of view were randomly selected. The microscopic images were analyzed using ImageJ software to measure villus height, crypt depth, and the villus height to crypt depth ratio (V/C) (15).

### 2.7 Microbial DNA extraction and sequencing of 16S rRNA genes

Based on comprehensive pharmacodynamic evaluations including growth performance parameters, serum biochemical indices, and intestinal HE staining analyses, the CON group, ATB group, and the most therapeutically effective TCYP dosage group were selected for 16S rRNA sequencing of cecal contents, which represent the intestinal segment with the highest microbial density in avian species.

The cecal content samples collected from broilers as described in Section 2.2 were subjected to microbial DNA extraction following the manufacturer’s protocol of the DNA extraction kit. To determine the final DNA concentration, RT-qPCR was used, and the integrity of the DNA was evaluated using agarose gel electrophoresis. The V3-V4 variable region of the 16S rRNA gene was amplified using primers 341F and 805R (341F: 5’-CCTACGGGNGGCWGCAG-3′; 805R: 5’-GACTACHVGGGTATCTAATCC-3′). The PCR amplicons were purified using the AMPure XP kit before to detection and quantification. To sequence the purified amplicons, the Illumina NovaSeq 6,000 platform was used.

Low-quality and chimeric sequences were filtered from the raw sequencing data to obtain high-quality clean data. The clean data were then denoised using DADA2 in Qiime2 to remove PCR amplification and sequencing errors, generating an amplicon sequence variant (ASV) abundance table for subsequent alpha diversity, beta diversity, species composition, and LEfSe analysis (16).

### 2.8 Metabolomics analysis

Metabolites were extracted from the contents of the chicken’s cecum, and then the extraction solvent was added, bead-beating, and ultrasonication were performed. After centrifugation, the supernatant was collected and lyophilized for examination. A Vanquish Flex UPLC system with an ACQUITY UPLC T3 column (100 mm x 2.1 mm, 1.8 μm) was used for the separation. Acetonitrile (B) and 5 mmol/L ammonium acetate-acetic acid aqueous solution (A) were used as mobile phases, and the column temperature was kept at 40°C. The flow rate was 0.35 mL/min. In both positive and negative ion modes (±3,800/3400 V), mass spectrometric detection was carried out using an Orbitrap Exploris 120 high-resolution mass spectrometer. The ion source temperature was set at 350°C, and the gas parameters were set as sheath gas 1, auxiliary gas 15, and nebulizer gas 50. After ten injections, quality control samples were examined to guarantee the accuracy of the results.

### 2.9 WGCNA analysis based on the GEO database

The GEO database provided the gene expression information for chicken gut health from the GSE94095 dataset. R 4.4.2 was used for data standardization, and the WGCNA R program was used for co-expression network analysis of the GSE94095 dataset (17). By choosing a suitable soft threshold, a weighted connection network was built, and dynamic tree cut analysis was used to find gene clusters. Modules exhibiting a substantial link with Intestinal health (correlation coefficient > 0.7, *p* < 0.05) were chosen after examining the statistical relationship between module eigengenes (MEs) and phenotypic features.

### 2.10 Construction of protein–protein interaction network

The SwissTargetPrediction^1^ database was used to predict chemical targets using TCYP chemicals derived from UHPLC-QE-MS analysis. The GeneCards database was used to find targets linked to illnesses of the chicken intestine.^2^ The intersection of pharmaceutical targets, disease-related genes, and significant genes from the WGCNA modules was calculated in order to identify the intersecting gene set. Using the STRING database,^3^ a protein–protein interaction network was constructed with a confidence score criterion of ≥ 0.4. Using Cytoscape 3.7.2, the network was visualized (18).

### 2.11 Gene ontology and KEGG pathway analysis

The clusterProfiler package in R was used to perform GO and KEGG pathway enrichment analyses on the intersection gene set. A *p*-value of less than 0.05 was established as the significance criterion. For visualization, loops of KEGG pathway and GO enrichment analyses were produced.

### 2.12 Reverse transcription quantitative PCR

After extracting RNA from chicken jejunal tissues, reverse transcription was performed. RT-qPCR was then used to determine the expression levels of *MMP1*, *PDPK1*, *PPARG*, *Bax*, and *Bcl-2*, using *β-actin* as an internal reference. The 2^-ΔΔCt method was used to determine the levels of gene expression. Table 3 contains the primer sequences for the target genes.

**Table 3:** The primer sequences of *MMP1*, *PDPK1*, *PPARG*, *Bad*, *Bcl-2*.

Gene	Sequences
*β-actin*	F: ACACCCACACCCCTGTGATGAA
	R: TGCTGCTGACACCTTCACCATTC
*MMP1*	F: TGCCCATCGAGCTCTACAAC
	R: TCCCGGAGGAAGTAGTAGCC
*PDPK1*	F: CCAGCAGCCACCTGTATGAT
	R: TGCCACAGGTGGAAATGACA
*PPARG*	F: TGACAGCGCCAGAGATTACA
	R: CATCCATCGCAGACAGATCCA
*Bcl-2*	F: ATCGTCGCCTTCTTCGAGTT
	R: ATCCCATCCTCCGTTGTCCT
*Bax*	F: ACTCTGCTGCTGCTCTCCTCTC
	R: ATCCACGCAGTGCCAGATGTAATC

### 2.13 Statistical analysis

Statistical analyses were performed using individual broilers as independent experimental units (biological replicates, *n* = 6 per group). The sample size was determined through *a priori* power analysis using G*Power software (version 3.1.9.7). Based on a one-way ANOVA model with an effect size *f* = 0.25, *α* = 0.05, and power = 0.95, the calculated actual power was 95.21%. Continuous data are presented as mean ± standard deviation. All statistical analyses were conducted using GraphPad Prism 6 and SPSS 26.0 software. Comparisons between two groups were performed using Student’s t-test, while multiple group comparisons were analyzed by one-way ANOVA. A *p*-value < 0.05 was considered statistically significant.

## 3 Results

### 3.1 Effect of TCYP on growth performance in broilers

**Figure 1:**
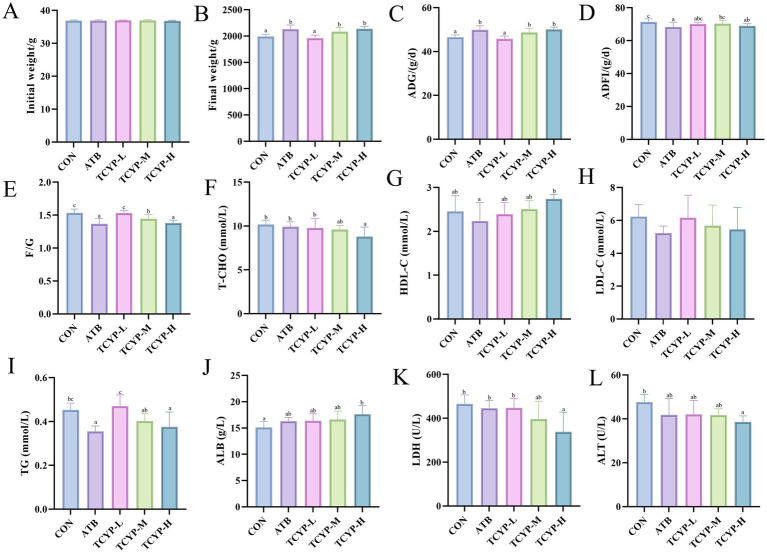
Effects of TCYP on growth performance and serum biochemical parameters in broilers. **(A)** Initial weight; **(B)** Final weight; **(C)** ADG; **(D)** ADFI; **(E)** F/G; **(F)** T-CHO; **(G)** HDL-C; **(H)** LDL-C; **(I)** TG; **(J)** ALB; **(K)** LDH; **(L)** ALT. Data were shown as means ± standard deviations (*n* = 6). Values with the same or no letter superscripts mean no significant difference (*p* > 0.05), while with different letter superscripts mean significant difference (*p* < 0.05).

Figures 1A–E demonstrate that compared with the CON group, low-dose TCYP showed no significant effects on the FBW and ADG of broilers (*p* > 0.05). In contrast, the ATB group, TCYP-M group, and TCYP-H group all significantly enhanced broiler FBW and ADG (*p* < 0.05). ADFI was significantly impacted by the ATB and high-dose TCYP groups (*p* < 0.05). The F/G ratio was considerably lower in the ATB and medium-high-dose TCYP groups than in the CON group (*p* < 0.05). This suggests that both ATB and medium-high doses of TCYP may enhance broiler growth performance by improving feed utilization efficiency.

### 3.2 Effect of TCYP on serum biochemical parameters in broilers

Figures 1F–L, compared to the CON group, the high-dose TCYP treatment significantly affected the levels and activities of T-CHO, TG, ALB, LDH, and ALT in the serum of broilers (*p* < 0.05). Compared to the CON group, the high-dose TCYP group showed a significant reduction in T-CHO, TG, and LDH levels, as well as ALT activity, along with a marked increase in ALB content (*p* < 0.05). In contrast, no significant changes were observed in the medium-and low-dose TCYP groups relative to the CON group (*p* > 0.05). In contrast to the CON group, a significant reduction was exclusively observed in TG concentrations within the ATB group (*p* < 0.05). Compared to the CON group, there were no significant differences in HDL-C and LDL-C levels across the groups (*p* > 0.05).

### 3.3 Effect of TCYP on intestinal morphology in broilers

**Figure 2:**
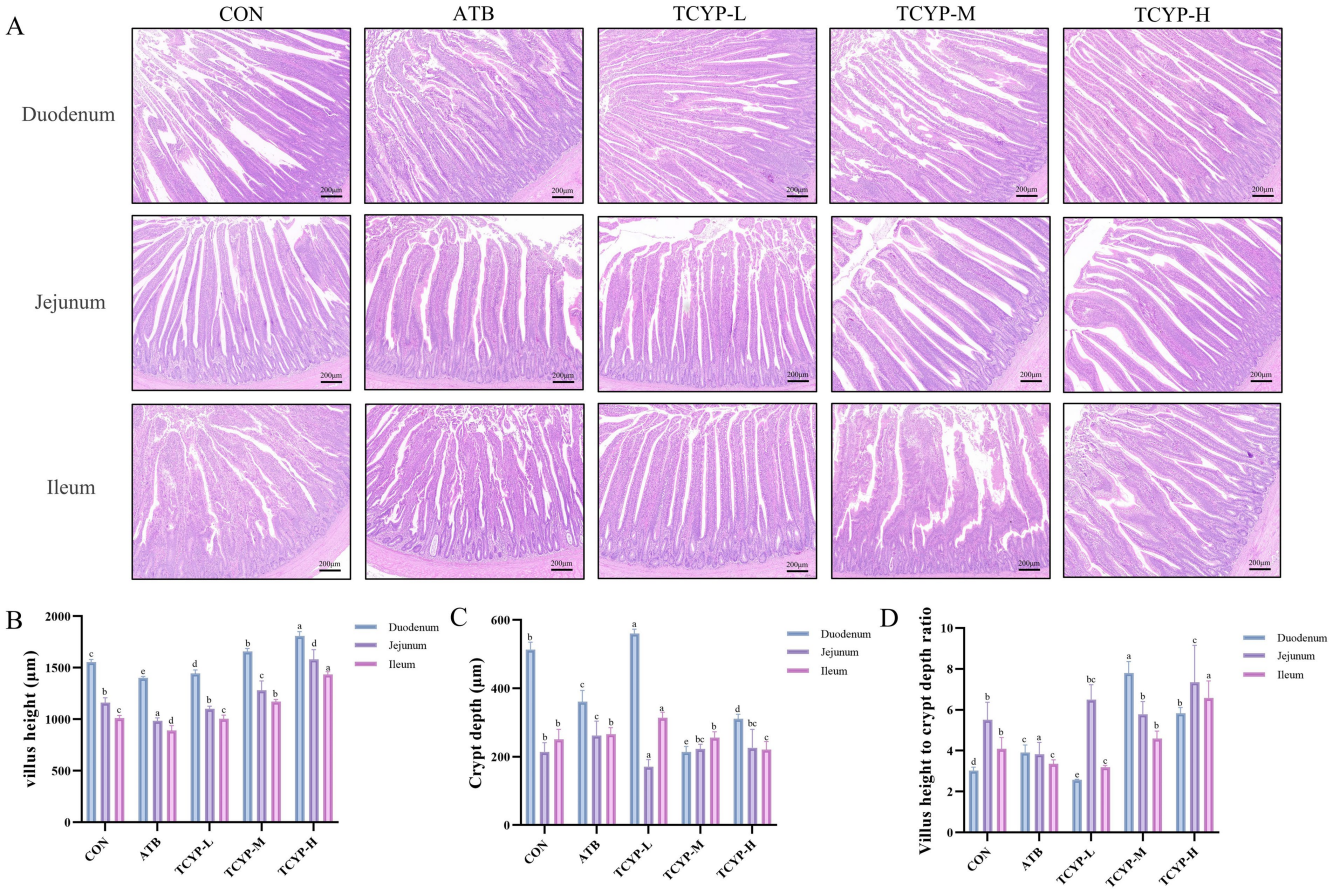
Effects of TCYP on the morphology of duodenum, jejunum, and ileum in broilers. **(A)** HE staining of duodenal, jejunal, and ileal tissues (scale bar: 100 μm); **(B)** Villus length; **(C)** Crypt depth; **(D)** Villus length to crypt depth ratio. Data were shown as means ± standard deviations (*n* = 6). Values with the same or no letter superscripts mean no significant difference (*p* > 0.05), while with different letter superscripts mean significant difference (*p* < 0.05).

Compared to the CON group, moderate and high dosages of TCYP significantly increased the villus height in the duodenum, jejunum, and ileum of broilers (*p* < 0.05), as Figure 2 illustrates. Compared to the CON group, the TCYP-H group showed a considerable reduction in crypt depth in the duodenum and ileum, along with a significantly higher villus height to crypt depth ratio in all three intestinal segments (*p* < 0.05). Furthermore, the antibiotic-containing diet induced significant morphological alterations in the duodenum, jejunum, and ileum of broilers compared to the CON group, manifesting as decreased villus height in all three segments and a markedly lower villus height to crypt depth ratio in the jejunum and ileum (*p* < 0.05).

### 3.4 Effect of TCYP on gut microbiota in broilers

**Figure 3:**
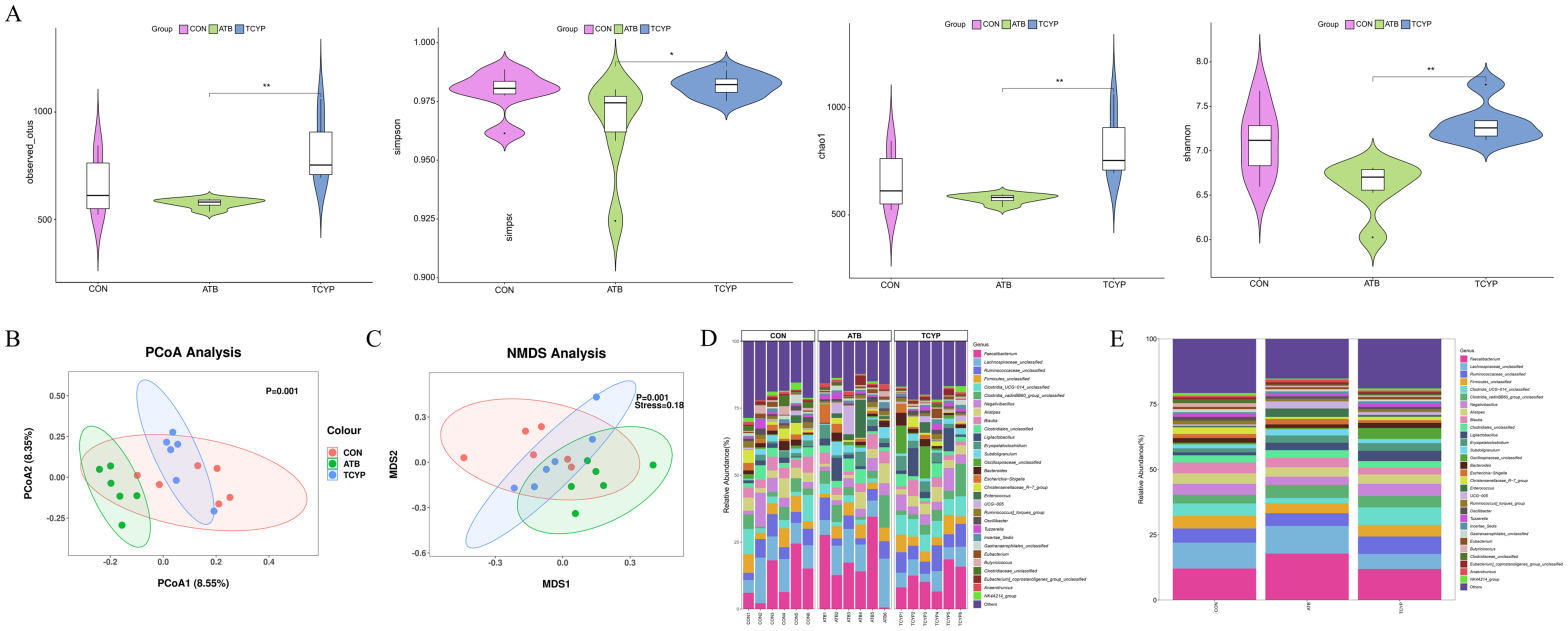
Effect of TCYP on gut microbiota in broilers. **(A)** Alpha diversity analysis; **(B)** PCoA analysis; **(C)** NMDS analysis; **(D)** Genus-level community composition analysis between samples; **(E)** Genus-level community composition analysis between groups. Data were shown as means ± standard deviations (*n* = 6). * indicates a significant difference between the TCYP and ATB groups (*p* < 0.05), ** indicates a highly significant difference between the TCYP and ATB groups (*p* < 0.01).

The detected OUT, Chao1, Shannon, and Simpson indices of the cecal microbiota did not differ statistically significantly between the ATB and TCYP groups and the CON group (*p* > 0.05), per the results of the Alpha diversity analysis in Figure 3A. When high-dose TCYP was added to the broiler diet, the cecal microbiota OUT, Chao1, Shannon, and Simpson indices were significantly higher than those in the ATB group (*p* < 0.05). The results indicate that supplementation with 1,500 mg/kg TCYP not only promotes growth performance in broilers but also preserves the *α*-diversity of gut microbiota, thereby sustaining the ecological homeostasis of cecal microbial ecosystems.

While the CON group overlapped with the other groups (*p* < 0.01), the TCYP group’s coordinates differed considerably from the ATB group’s (PCoA analysis, Figure 3B). 8.55% of the variation between the treatment groups was explained by principal coordinate 1 (PCoA1), and 8.35% by principal coordinate 2 (PCoA2). For the discrepancies, the NMDS analysis (Stress = 0.18), too, produced trustworthy findings (Figure 3C). The cecal microbiota composition analysis at the genus level (Figure 3D,E) showed that *Faecalibacterium*, *Lachnospiraceae_unclassified*, *Ruminococcaceae_unclassified*, and *Firmicutes_unclassified* were the dominant species. The average proportion of *Faecalibacterium* in the CON group was 12.08%, which increased to 17.81% in the ATB group and decreased to 11.95% in the TCYP group. The average proportion of *Lachnospiraceae_unclassified* in the CON group was 9.90%, which slightly increased to 10.57% in the ATB group and decreased to 5.68% in the TCYP group. The average proportion of *Ruminococcaceae_unclassified* in the CON group was 5.45%, which decreased to 4.87% in the ATB group and increased to 6.72% in the TCYP group. The average proportion of *Firmicutes_unclassified* in the CON group was 4.86%, which decreased to 3.92% in the ATB group and further decreased to 4.45% in the TCYP group.

### 3.5 Effect of TCYP on cecal metabolites

**Figure 4:**
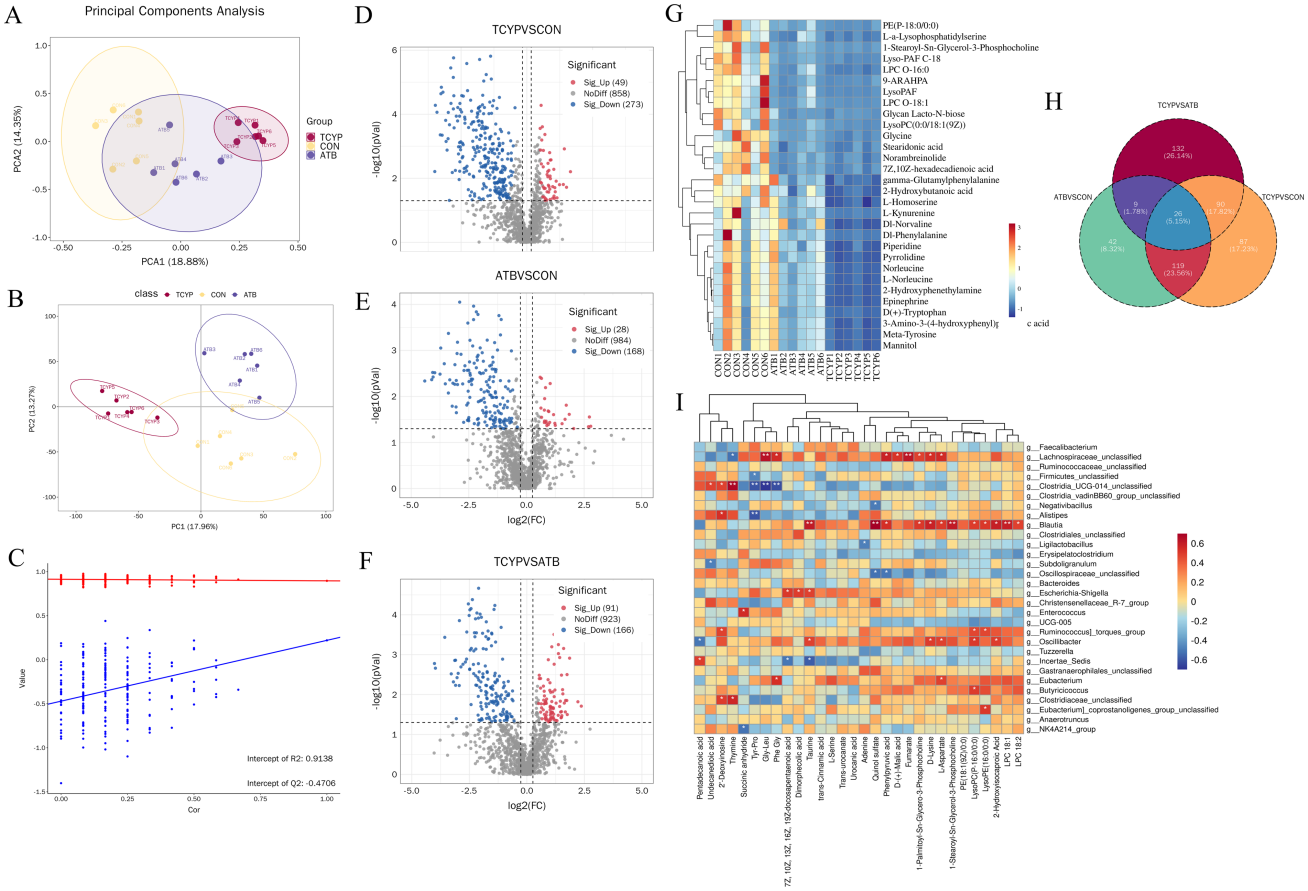
Cecal metabolomics analysis. **(A)** PCA analysis; **(B)** PLS-DA analysis; **(C)** Permutation test analysis; **(D)** Volcano plot of differential metabolites between TCYP and CON; **(E)** Volcano plot of differential metabolites between ATB and CON; **(F)** Volcano plot of differential metabolites between TCYP and ATB; **(G)** Heatmap of the top 30 differential metabolites; **(H)** Venn diagram of differential metabolites between groups; **(I)** Spearman correlation analysis between differential microbial taxa and differential metabolites in the cecum. Data are presented for a sample size of *n* = 6.

As shown in Figure 4A, differential metabolites were collected under both positive and negative ion modes, and the PCA plot illustrates the overall distribution of the dataset. In the PCA model, QC samples tightly clustered, indicating the stability and reliability of the experimental data. Meanwhile, a separation trend was observed among the three groups of broilers in the PCA plot, particularly between the CON and TCYP groups, suggesting potential metabolic differences between groups. This separation trend was further validated by the PLS-DA model (Figure 4B). As seen by Figure 4C, the permutation test verified that the PLS-DA model was not overfitted after 200 rounds of 7-fold cross-validation.

Based on FC ≥ 1.2 or FC ≤ 1/1.2, *p* value < 0.05, VIP ≥ 1, differential metabolites after ATB and TCYP treatments were identified. Comparison of the TCYP and CON groups showed 322 metabolites with significant changes, including 49 metabolites upregulated in the TCYP group, such as Esculin, Lysophosphatidylglycerol O-13:1, and D-mannose 6-phosphate, and 273 metabolites downregulated in TCYP, including L-Malic acid, (Z)-13-Docosenamide, and Diacylglycerol 8:0_16:3 (Figure 4D). Comparison between the ATB and CON groups showed 196 metabolites with significant changes, including 28 metabolites upregulated in TCYP, such as Cadaverine, Voacristine, and N-Acetylputrescine, and 168 metabolites downregulated, including Lysophosphatidylcholine O-18:2, Monoacylglycerol 19:0, and Trans-urocanate (Figure 4E). Comparison between the TCYP and ATB groups revealed 257 metabolites with significant changes, including 91 metabolites upregulated in TCYP, such as Lysophosphatidylglycerol O-13:1, Monogalactosyldiacylglycerol O-16:2_24:5, and 2-Methyladipic acid, and 166 metabolites downregulated in TCYP, including Glutamic acid, N-Acetyl-L-leucine, and Octadecanamide (Figure 4F). Figure 4G displays a heatmap of the top 30 differential metabolites, with blue denoting relatively low abundance and red denoting relatively high abundance. The distinct and similar effects of the various treatments on the metabolites are revealed by the Venn diagram of differential metabolites between the TCYP, ATB, and CON groups, which displays overlapping and particular changes in metabolites (Figure 4H).

Metabolites and gut microbiota were shown to be highly associated, according to the results of Spearman correlation analysis (Figure 4I). Notably, *Lachnospiraceae_unclassified* showed a significant positive correlation with Gly-Leu, Fumarate, Phenylpyruvic acid, L-Aspartate, Phe Gly, D-Lysine, and D-(+)-Malic acid (*r* > 0.5, *p* < 0.05) and a significant negative correlation with Thymine (*r* < −0.5, *p* < 0.05), indicating that this microbiota may be involved in the regulation of amino acid metabolism, energy metabolism, and organic acid synthesis.

### 3.6 Co-expression network construction and module identification

**Figure 5:**
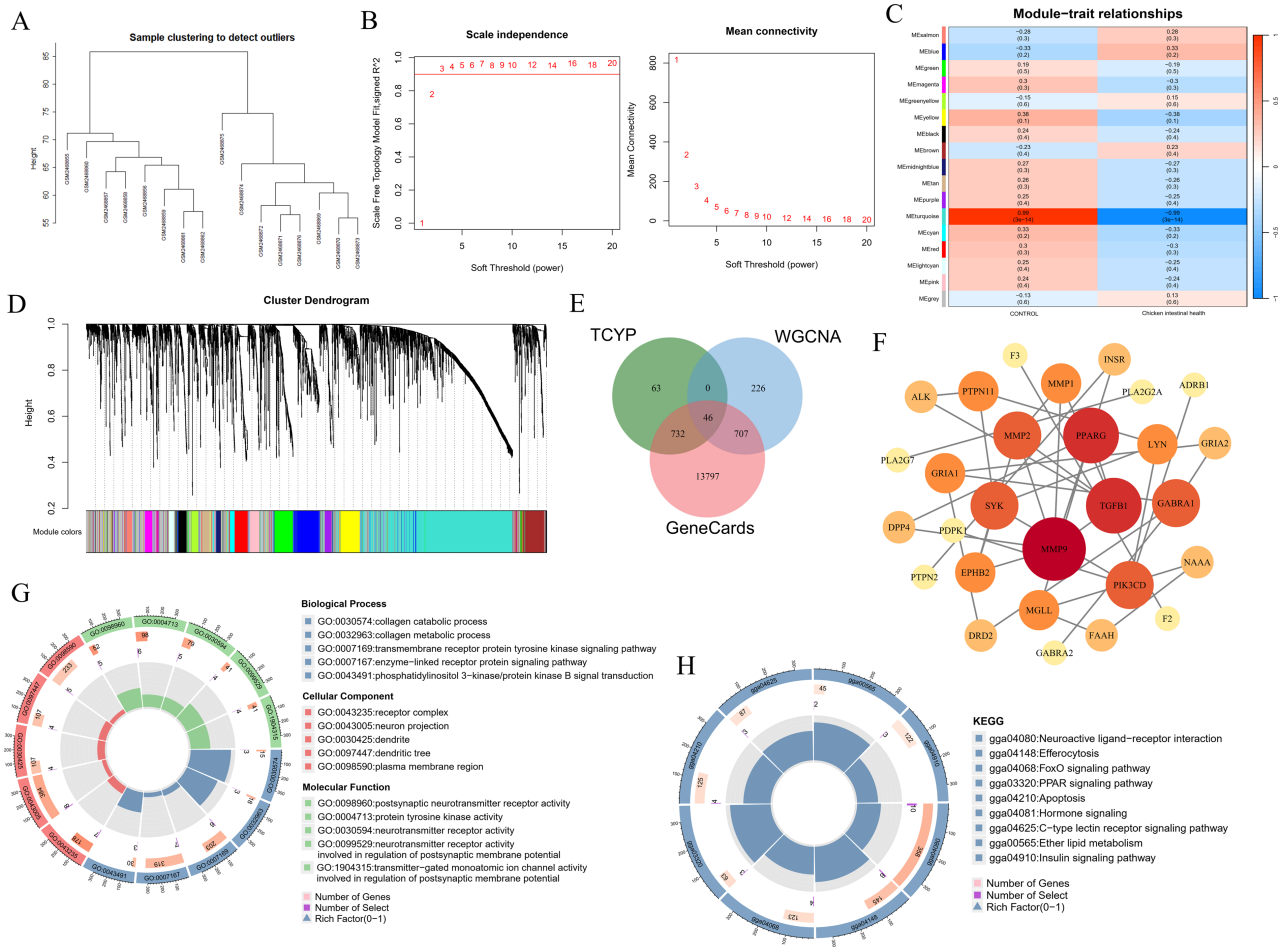
Key gene modules and molecular mechanisms of TCYP regulation of Chicken intestinal health. **(A)** Sample clustering dendrogram; **(B)** Soft threshold power selection; **(C)** Representative heatmaps of correlations between modules; **(D)** The clustering tree diagram of the co-expression module. Different colors represent different co-expression modules; **(E)** Venn diagram of overlapping genes between the MEturquoise module genes, chicken intestinal health-related disease genes, and TCYP-related targets; **(F)** Protein–protein interaction network; **(G)** Gene Ontology analysis; **(H)** KEGG pathway enrichment analysis.

To identify key gene modules associated with chicken intestinal health, this study utilized the WGCNA algorithm based on the GSE94095 dataset from the GEO database. 16 samples from the control and healthy chicken intestine groups were used to create a gene co-expression network (Figure 5A). Following the computation of the variance of gene expression, the top 25% of genes with the largest variation were chosen for further examination. By optimizing the soft thresholding power (soft thresholding power = 14) and combining hierarchical clustering with the dynamic tree-cutting algorithm, a gene co-expression network was successfully built, identifying 17 unique gene modules (Figures 5B–D). Among these, the MEturquoise module showed a strong correlation with the chicken intestinal health phenotype (correlation coefficient = 0.99, *p* < 0.001), suggesting that this module may play a key role in regulating chicken intestinal health.

The compounds derived from UHPLC-QE-MS analysis were entered into the SwissTargetPrediction database for target prediction in order to further investigate the important genes implicated in the treatment of chicken intestinal health by TCYP. This resulted in 841 compound-related targets. In the meanwhile, 15,282 targets linked to intestinal health in chickens were predicted using the GeneCards database. 46 key targets were chosen by examining the interaction of medication and disease targets with important genes in the MEturquoise module. To create a network of protein–protein interactions, these intersecting targets were then entered into the STRING database. TGFB1, PPARG, and MMP9 were selected as core targets based on the Degree value (Figures 5E,F).

### 3.7 Gene ontology (GO) and KEGG pathway analysis

Additional GO and KEGG pathway analyses were performed on the 46 significant genes using the R program. The greatest levels of cellular component (CC) enrichment were found in the plasma membrane region, receptor complex, and neuron projection, according to the GO analysis findings. The main molecular functions (MF) were endopeptidase activity, protein kinase activity, and protein tyrosine kinase activity. Biological processes (BP) were closely related to the regulation of cell population proliferation, the enzyme-linked receptor protein signaling pathway, and the transmembrane receptor protein tyrosine kinase signaling pathway (Figure 5G).

KEGG pathway enrichment analysis revealed that the FoxO signaling pathway and PPAR signaling pathway might be key pathways through which TCYP influences chicken intestinal health. These results provide important clues for revealing the molecular mechanisms by which TCYP regulates chicken intestinal health (Figure 5H).

### 3.8 TCYP activates PPAR pathway-related gene expression in the jejunum of broilers

**Figure 6:**
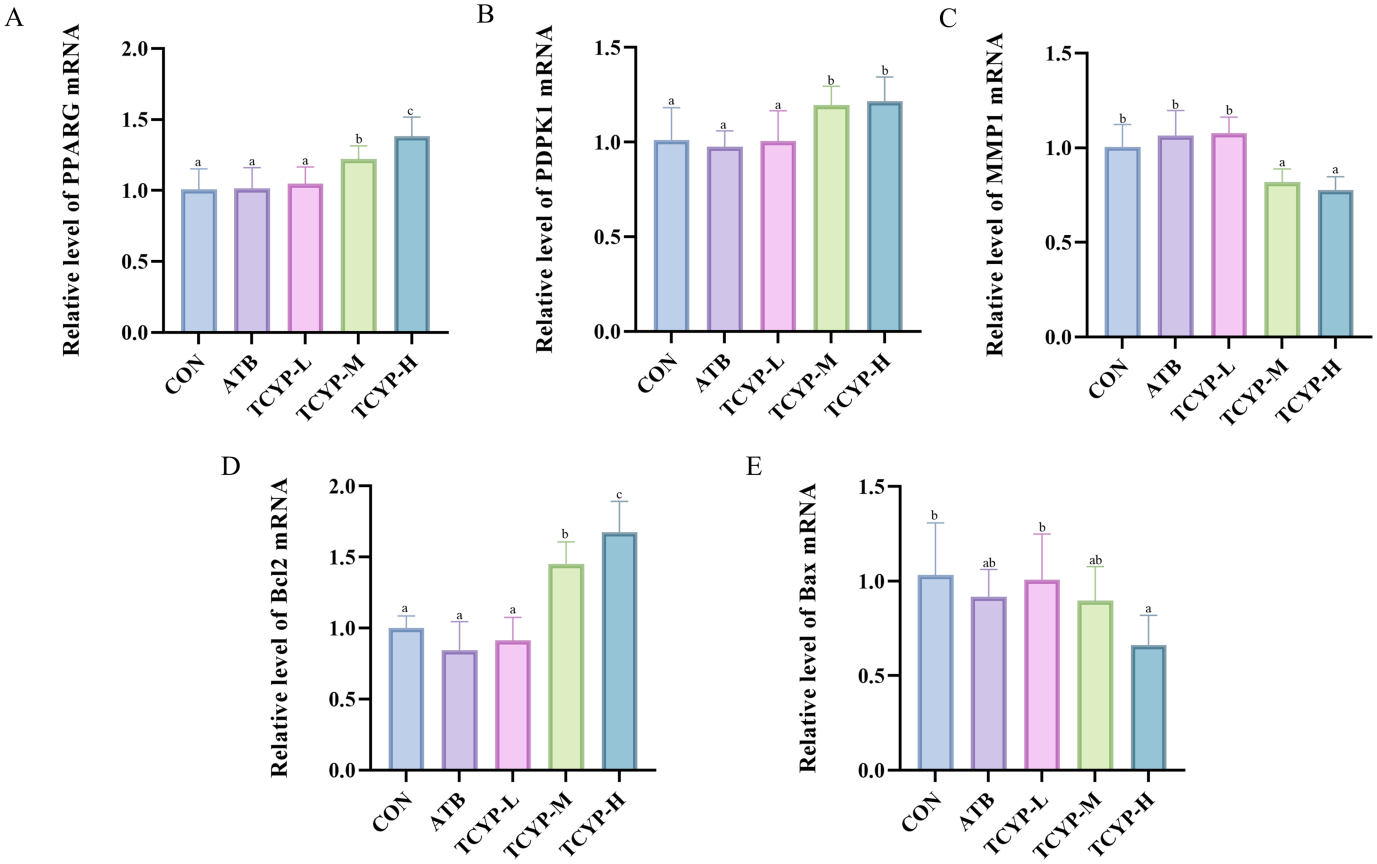
Effects of TCYP on gene expression of **(A)** *PPARG*, **(B)** *PDPK1*, **(C)** *MMP1*, **(D)** *Bcl2* and **(E)** *Bax* in the jejunum of broilers. Data were shown as means ± standard deviations (*n* = 6). Values with the same or no letter superscripts mean no significant difference (*p* > 0.05), while with different letter superscripts mean significant difference (*p* < 0.05).

As illustrated in Figure 6, we used real-time PCR to measure the expression levels of *PPARG* and its downstream genes. The ATB group’s jejunum showed no appreciable alterations in gene expression when compared to the CON group. *PPARG*, *PDPK1*, and *Bcl2* gene expression in the TCYP group considerably increased in a dose-dependent manner (*p* < 0.05) as TCYP concentration rose, but *MMP1* and *Bax* gene expression dramatically reduced in a dose-dependent manner (*p* < 0.05). The results demonstrate that TCYP potentially ameliorates intestinal barrier integrity through PPARG-mediated regulation of proliferative and apoptotic processes. This mechanistic insight is consistent with the histomorphological improvements observed in TCYP-H treated broilers, substantiating the hypothesis that TCYP’s enteroprotective efficacy stems from coordinated multi-target modulation.

## 4 Discussion

The water extract of TCYP contains various active components, including Succinic acid, Prim-O-glucosylcimifugin, Linarin, and others. These components have been widely reported in medical literature for their anti-inflammatory, antioxidant, and therapeutic effects on various gastrointestinal diseases (19). In recent years, the application of TCYP has gained increasing popularity in animal husbandry practices. According to a study by Zheng et al. (10) YuPingFeng polysaccharides can enhance chick growth performance by controlling gut microbiota, antioxidant balance, and serum immunity. The results of this experiment show that adding medium to high doses of TCYP to the diet of 1–42 day-old broilers significantly increased their average daily weight gain and reduced the feed-to-weight ratio. Serum biochemical markers including T-CHO and TG are closely associated with lipid metabolism. Specifically, TG serves as the primary form of lipid storage in hepatocytes, while T-CHO predominantly functions in systemic lipid transport (20). For hepatic function assessment, LDH and ALT are well-established biomarkers (21). LDH activity reflects the extent of tissue damage, with decreased levels indicating attenuated cellular necrosis and injury (22). As a hepatocyte-specific enzyme, ALT exhibits elevated serum levels when cellular membrane permeability increases (23). The observed reduction in serum LDH and ALT activities demonstrates that TCYP effectively enhances hepatic function in broilers, thereby optimizing lipid metabolism to support growth performance and energy provision. In addition, ALB is the major protein in blood and not only helps maintain the colloidal osmotic pressure but also transports various substances (24). Studies have shown that an increase in ALB levels may positively affect immune function and nutritional status (25). The alterations in these serum biochemical parameters indicate that TCYP may enhance the physiological status of broilers by regulating lipid metabolism and improving hepatic function.

In the small intestine, the morphological markers of villus height, crypt depth, and villus/crypt ratio are often used to precisely evaluate the functional and overall health of chickens. The structure of the intestinal microbiota, the crypt depth ratio, and the length of the villus in the duodenum and jejunum were all considerably enhanced by adding 10 g/kg of Rumex nepalensis to broiler diets, according to Banday et al. (26). The villus length and villus height to crypt depth ratio in the jejunum were greatly raised by adding 1,500 mg/kg of TCYP to the diet, but the same was dramatically lowered by adding ATB. The findings suggest that TCYP likely improves nutrient assimilation by optimizing intestinal architecture. The comparative advantage of TCYP over ATB in preserving gut morphological integrity provides compelling evidence for its application as a sustainable antibiotic alternative in poultry production.

The 16S rRNA sequencing results indicated reduced *α*-diversity in the ATB group. Compared with the ATB group, dietary TCYP supplementation increased the α-diversity indices of broiler intestinal microbiota, demonstrating that TCYP can enhance broiler growth performance while maintaining intestinal microecological balance. Previous studies have confirmed that coexisting components in traditional Chinese medicine compounds can enhance intestinal nutrient absorption by improving solubility and increasing the permeability of intestinal epithelial cell membranes, suggesting that TCYP may improve the growth performance of broilers through this mechanism (27). YuPingFeng polysaccharide supplementation in feed has been shown by Yin et al. to enhance chick development performance by controlling gut microbiota, antioxidant balance, and serum immunology (14). *Faecalibacterium* and *Lachnospiraceae_unclassified* were more abundant in this experiment’s gut microbiota composition analysis at the genus level when ATB was added to the diet than in the CON group, whereas *Ruminococcaceae_unclassified* and *Firmicutes_unclassified* were less abundant. In contrast, after adding TCYP to the diet, the relative abundance of *Faecalibacterium*, *Lachnospiraceae_unclassified*, and *Firmicutes_unclassified* decreased, while the relative abundance of *Ruminococcaceae_unclassified* increased. The findings indicate that TCYP potentially ameliorates the gut microenvironment via selective regulation of particular microbial taxa, corroborating its established pharmacological efficacy in promoting intestinal health. *Thesium chinense*, known as “plant antibiotic” in traditional Chinese medicine, has been widely verified for its inhibitory activity against harmful bacteria like *Staphylococcus aureus* (28). Therefore, while *Thesium chinense* may inhibit harmful bacteria, it could also have some inhibitory effect on beneficial bacteria, leading to a slight decrease in the abundance of dominant gut microbiota. Pseudoflavonifractor, *Faecalibacterium*, Ruminococcus species, and many genera under the Firmicutes phylum are closely related to the production of butyrate, which plays a key role in repairing and enhancing the barrier function of intestinal epithelial cells. Butyrate, as a short-chain fatty acid, not only provides energy for colonocytes but also contributes to maintaining gut health by strengthening the intestinal epithelial integrity and modulating the immune response (29–31). It has been shown that butyrate can support intestinal development, inhibit intestinal pathogens, reduce pro-inflammatory cytokines, and promote the activation of regulatory T cells (Treg) (32, 33). Through the *β*-hydroxy-β-methylbutyrate-CoA pathway, butyrate may promote lipid synthesis from acetyl-CoA or ketone bodies, potentially leading to weight gain (34). However, the effects of JYPF on intestinal butyrate levels in broilers require further verification.

Endogenous metabolites and the gut microbiota are intimately associated. The results of PCA and PLS-DA in this experiment showed significant metabolic differences among the groups. A total of 26 differential metabolites were identified as the common differences between the three groups, primarily consisting of lipids, amino acids, and organic acids. Abnormal glycerophospholipid metabolism is often associated with the development of various intestinal diseases. Phosphatidylcholine can hydrolyze into lysophosphatidylcholine to activate immune cells and participate in inflammatory responses (35). In addition, the decrease in serum indicators such as T-CHO and TG in this experiment further emphasizes the important role of lipid metabolism in intestinal diseases. The metabolomics results showed that after the addition of TCYP to the diet, the levels of phospholipid components such as L-Malic acid, (Z)-13-Docosenamide, and Diacylglycerol 8:0_16:3 were downregulated, while components such as Esculin, Lysophosphatidylglycerol O-13:1, and D-mannose 6-phosphate were upregulated. This suggests that regulating lipid metabolism may be a key mechanism through which TCYP improves chicken gut health. Through regulating the expression of the miR-181 family in white adipocytes, Virtue et al. discovered that tryptophan-derived compounds generated by the gut microbiota affect insulin sensitivity, energy expenditure, and host fat accumulation in mice (36). The Spearman correlation analysis between *Lachnospiraceae_unclassified* and various amino acids and organic acids indicates that this genus may play a significant role in amino acid metabolism and energy metabolism. Especially the positive correlation with Fumarate and L-Aspartate suggests that *Lachnospiraceae_unclassified* may be involved in key metabolic steps of the tricarboxylic acid cycle, thereby affecting energy production and cellular metabolic balance (37). Thymine, as an important precursor for DNA synthesis, its reduced abundance may be related to the regulation of cell proliferation and repair processes (38). The negative correlation between *Lachnospiraceae_unclassified* and Thymine may reflect the regulatory role of this bacterial genus in nucleotide metabolism.

We mined the chicken gut health dataset from the GEO database and conducted R analysis, identifying core targets such as *MMP9*, *TGFB1*, and *PPARG*. We also found that the PPAR signaling pathway may be a potential pathway through which TCYP improves chicken gut health. The PPAR family plays a crucial role in cell signaling, metabolic regulation, inflammation, and gene expression (39). PPARγ stimulates the upregulation of FABP, which in turn facilitates the absorption and storage of long-chain fatty acids in cells (40). Activation of PPARγ leads to the activation of PDPK1, which, through phosphorylation of Akt, exerts its pro-survival effects on cells (41). This activation further triggers Bcl2 and downregulates Bax, thereby alleviating cell apoptosis. Ye et al. used Chrysosplenosides I and A to treat fruit flies and aging mice, and found that these compounds activated the PPAR signaling pathway while inhibiting the EGFR signaling pathway to restore intestinal stem cell aging (42). We further validated these findings using real-time PCR and found that TCYP significantly upregulated the mRNA expression of *PPARG*, *PDPK1*, and *Bcl2* in the jejunal tissue, while significantly downregulating the mRNA expression of *MMP1* and *Bax*.

In contrast to antibiotics which primarily function through pathogen suppression, TCYP exerts its beneficial effects via multi-target and multi-pathway regulatory mechanisms to enhance intestinal health (43). While antibiotics can rapidly reduce pathogenic bacterial loads, prolonged administration often leads to microbiota dysbiosis and increased antibiotic resistance (44). In comparison, accumulating evidence has demonstrated TCYP’s capacity to modulate gut microbial composition (45). Notably, our findings reveal that although both TCYP and antibiotics improve broiler growth performance, TCYP demonstrates superior efficacy in maintaining intestinal barrier integrity, preserving microbial homeostasis, and regulating lipid metabolism. Despite its promising potential, TCYP still presents several challenges compared to conventional antibiotics. For instance, the multi-component nature of herbal formulations leads to complex active ingredients, which may vary between batches due to differences in geographical origin, harvest season, and processing methods. Additionally, the high cost of raw medicinal materials and the complexity of extraction processes may hinder large-scale industrial production.

However, this study has several limitations that warrant discussion. First, although metabolomic analysis identified multiple differential metabolites, the lack of functional validation experiments makes it difficult to definitively establish their specific contributions to TCYP-mediated intestinal health improvement. To address this, we plan to employ *in vitro* cell models and animal intervention studies, where exogenous supplementation of key metabolites (e.g., L-malic acid, esculin) will be used to assess their regulatory effects on intestinal barrier function, inflammation, and metabolism. Second, while bioinformatics analysis suggests that the PPAR signaling pathway may mediate TCYP’s beneficial effects on broiler gut health, the absence of commercially available antibodies for certain key proteins (e.g., MMP1, PDPK1) has hindered protein-level validation. To overcome this limitation, we will utilize targeted proteomic approaches for quantitative protein analysis in future investigations. Furthermore, integrating organoid culture systems and single-cell sequencing could provide higher spatiotemporal resolution in elucidating TCYP’s effects on intestinal epithelial differentiation and host-microbiota crosstalk. While the 42-day experiment encompassed the full growth cycle of broiler chickens, further multi-generational studies are necessary to fully assess the long-term stability and safety of TCYP as a sustainable antibiotic alternative. Finally, this study demonstrates that TCYP exhibits clear dose-dependent effects on broiler growth performance, serum biochemical parameters, intestinal health, and the PPAR signaling pathway. However, the impact of higher TCYP doses on broiler physiological functions and the underlying mechanisms require further systematic dose–response studies for comprehensive elucidation. These refinements will facilitate a more comprehensive understanding of TCYP’s multi-component synergistic mechanisms and support its standardized application in poultry production.

## 5 Conclusion

The results of this study demonstrate that dietary supplementation with 1,500 mg/kg TCYP significantly enhances broiler growth performance and serum biochemical parameters compared to lower doses (500 mg/kg and 1,000 mg/kg). Furthermore, 1,500 mg/kg TCYP improves intestinal morphology, stabilizes gut microbiota composition, and promotes intestinal health by modulating lipid and amino acid metabolism. Bioinformatics and gene expression analyses suggest that the PPAR signaling pathway may play a key role in these effects, with TCYP dose-dependently regulating the mRNA expression of this pathway. These findings indicate that TCYP could be a candidate for further investigation as a potential alternative to feed antibiotics, though additional validation is required to confirm its efficacy and mechanisms.

## Data Availability

Publicly available datasets were analyzed in this study. This data can be found here: https://www.ncbi.nlm.nih.gov/geo/query/acc.cgi?acc=GSE94095; GSE94095.

## Footnotes

^1^ http://www.swisstargetprediction.ch/

^2^ https://www.genecards.org/

^3^ https://string-db.org/

## Glossary

TCYP: BaiRui YuPingFeng Powder
CON: control group
ATB: antibiotic group
TCYP-L: 500 mg/kg dose of TCYP
TCYP-M: 1,000 mg/kg dose of TCYP
TCYP-H: 1,500 mg/kg dose of TCYP
UHPLC-QE-MS: Ultra-high-performance liquid chromatography coupled with quadrupole-Orbitrap mass spectrometry
ADG: average daily gain
ADFI: average daily feed intake
F/G: feed-to-gain ratio
T-CHO: total cholesterol
TG: triglycerides
LDH: lactate dehydrogenase
ALT: alanine aminotransferase
ALB: albumin
PPAR: peroxisome proliferator-activated receptor
qPCR: Quantitative PCR
WGCNA: weighted gene co-expression network analysis
IBW: Initial body weight
FBW: final body weight
TC: total cholesterol
HDL-C: high-density lipoprotein cholesterol
LDL-C: low-density lipoprotein cholesterol
ASV: amplicon sequence variant
PCA: Principal Component Analysis
PCoA: Principal Coordinates Analysis
SD: standard deviation
GO: Gene Ontology
CC: cellular components
MF: molecular functions
BP: biological processes
