# Supplementary material for: Integrative multi-omics and bioinformatics analysis of the effects of BaiRui YuPingFeng Powder on intestinal health in broilers
Source: Front Vet Sci. 2025 Jun 18;12:1606531. doi: 10.3389/fvets.2025.1606531 (PMC12213368; doi:10.3389/fvets.2025.1606531)
Supplement: Supplementary file 1 [file Table_1.DOCX]

Supplementary Material

# UHPLC-QE-MS Analysis for Compound Identification

An ultra-high-performance liquid chromatography system, the Nexera UHPLC LC-30A, was used to conduct the investigation. After equilibrating the column (ACQUITY UPLC BEH Amide, 2.1 × 100 mm, 1.7 μm) with 98% mobile phase A, gradient elution was performed at a flow rate of 0.3 mL/min. With a Q Exactive HF-X mass spectrometer, analysis was carried out in both positive and negative ion modes. Both qualitative and quantitative analyses were performed on the raw data obtained from the UHPLC-QE-MS equipment in order to describe the chemical makeup and metabolic profile of the TCYP aqueous extract.

Secondary mass spectrometry was used to identify 52 chemicals in both positive and negative ion modes. Thirty-six compounds were found in the negative ion mode, while sixteen compounds were found in the positive ion mode. Various chemical classes, including sugars, glycosides, and organic acids, were represented in these molecules (Figure 1). Comprehensive details on the compounds are included in Table 2.

| Table 2 The compounds were identified by UHPLC-QE-MS analysis. | | | | | | |
| --- | --- | --- | --- | --- | --- | --- |
| NO | compounds | RT (min) | Adduct | m/z | ppm | Molecular Formula |
| 1 | l-6-deoxyascorbic acid | 12.67 | M+FA-H | 205.0354 | -0.14132 | C6H8O5 |
| 2 | maleylacetic acid | 12.51 | M-H | 157.0142 | -0.01006 | C6H6O5 |
| 3 | 4-butyrolactone | 12.44 | M-H | 85.02948 | -0.22083 | C4H6O2 |
| 4 | itaconic acid | 11.9 | M-H | 129.0194 | 0.196652 | C5H6O4 |
| 5 | 2h-pyran-2-one, 3-hydroxy- | 11.95 | M-H | 111.0088 | 0.204433 | C5H4O3 |
| 6 | methylmalonic acid | 10.51 | M-H | 117.0193 | -0.1462 | C4H6O4 |
| 7 | D-glycero-d-manno-octulose | 9.97 | M-H | 239.0772 | -0.13958 | C8H16O8 |
| 8 | 3-hydroxypyruvic acid | 9.68 | M-H | 103.0037 | -0.07614 | C3H4O4 |
| 9 | valienone | 9.2 | M+FA-H | 219.051 | 0.12188 | C7H10O5 |
| 10 | 2,3,4-trihydroxypentanoic acid | 8.68 | M+FA-H | 195.0511 | 0.449416 | C5H10O5 |
| 11 | arabinonate | 8.58 | M-H | 165.0405 | 0.276362 | C5H10O6 |
| 12 | pimelic acid | 8.44 | M+FA-H | 205.0718 | -0.04084 | C7H12O4 |
| 13 | 3,4-dihydroxybutanoic acid | 7.68 | M+FA-H | 165.0405 | 0.415 | C4H8O4 |
| 14 | 2-(2-hydroxyphenyl)ethanol | 6.92 | M+NH4 | 156.1016 | -1.8493 | C8H10O2 |
| 15 | threonate | 6.79 | M-H | 135.0299 | 0.254347 | C4H8O5 |
| 16 | piperidine | 6.26 | M+H | 86.09632 | -1.28834 | C5H11N |
| 17 | 4-hydroxycyclopent-2-en-1-one | 6.27 | M+FA-H | 143.035 | 0.213097 | C5H6O2 |
| 18 | 2-deoxy-d-ribofuranose | 5.57 | M+FA-H | 179.0561 | -0.00561 | C5H10O4 |
| 19 | 1,2,3,4,5-pentahydroxypentane | 5.42 | M-H, M+FA-H | 151.0612 | 0.23953 | C5H12O5 |
| 20 | Linarin | 5.18 | M+H, M+Na | 615.1683 | -4.51386 | C28H32O14 |
| 21 | dimethyl oxalate | 5.22 | M+FA-H | 163.0249 | 0.804915 | C4H6O4 |
| 22 | Succinic acid | 5.13 | M+FA-H | 163.0249 | 0.804915 | C4H6O4 |
| 23 | 3-O-ethyl ferulate | 5.29 | M+FA-H | 267.0872 | -0.99936 | C12H14O4 |
| 24 | gamma hydroxybutyric acid | 5.06 | M-H | 103.0401 | 0.146169 | C4H8O3 |
| 25 | 1-benzofuran-4-ol | 4.98 | M+FA-H | 179.035 | 0.128449 | C8H6O2 |
| 26 | hydroxybutyrate,l | 4.63 | M-H | 103.0401 | 0.154435 | C4H8O3 |
| 27 | methyl-pyruvate | 4.37 | M+FA-H | 147.03 | 0.671231 | C4H6O3 |
| 28 | 2-ethylpent-3-enoic acid | 4.22 | M+NH4 | 146.1174 | -1.32848 | C7H12O2 |
| 29 | isonicotinic acid | 4.18 | M-H | 122.0248 | 0.505972 | C6H5NO2 |
| 30 | Cimifugin | 4.22 | M+H | 307.1171 | -1.66901 | C16H18O6 |
| 31 | 2-butene-1,4-diol (trans) | 4.00 | M+FA-H | 133.0507 | 0.558939 | C4H8O2 |
| 32 | methyl-p-hydroxycinnamate | 3.85 | M+NH4 | 196.0965 | -1.66707 | C10H10O3 |
| 33 | Prim-O-glucosylcimifugin | 3.73 | M+H | 469.1697 | -1.62059 | C22H28O11 |
| 34 | 3, 5-dihydroxyphenethyl alcohol 3-O-β-D-glucopyranoside | 3.42 | M+NH4 | 334.149 | -2.14933 | C14H20O8 |
| 35 | methyl (2e)-oct-2-en-4,6-diynoate | 2.73 | M+FA-H | 193.0508 | 1.292861 | C9H8O2 |
| 36 | Phenylacrylic acid | 2.80 | M+FA-H | 193.0508 | 1.292861 | C9H8O2 |
| 37 | Scopolin | 2.43 | M+H | 355.1018 | -1.61867 | C16H18O9 |
| 38 | Atractylenolide iii | 2.18 | M+NH4 | 266.1747 | -1.62701 | C15H20O3 |
| 39 | 5-ethyl-2(5h)-furanone | 1.95 | M+FA-H | 157.0506 | 0.092166 | C6H8O2 |
| 40 | pinoresinol-4-O-D- glucoside | 1.73 | M+H-H2O, M+NH4 | 538.2275 | -1.39143 | C26H32O11 |
| 41 | 3-oxopropanoic acid | 1.37 | M-H | 87.00878 | 0.15181 | C3H4O3 |
| 42 | (4r,5s)-4,5-dihydroxy-3-methylcyclopent-2-en-1-one | 1.39 | M+FA-H | 173.0456 | 0.700613 | C6H8O3 |
| 43 | Hydroxyacetophenone | 3.76 | M+NH4 | 154.086 | -1.8312 | C8H8O2 |
| 44 | 3,4-di-o-caffeoylquinic acid | 1.28 | M+H | 517.1332 | -1.61538 | C25H24O12 |
| 45 | (2e)-pent-2-en-1-ol | 1.13 | M+FA-H | 131.0714 | 0.712284 | C5H10O |
| 46 | 6-[(2r)-butan-2-yl]-3-ethyl-4-hydroxypyran-2-one | 0.93 | M+FA-H | 241.1083 | 0.558082 | C11H16O3 |
| 47 | Calycosin | 0.96 | M+H | 285.0752 | -1.90196 | C16H12O5 |
| 48 | psoralen | 2.5 | M-H | 185.0229 | -2.22174 | C11H6O3 |
| 49 | salicyclic acid | 0.74 | M-H | 137.0245 | 0.446004 | C7H6O3 |
| 50 | ethylbenzene | 0.74 | M-H | 105.071 | 0.63146 | C8H10 |
| 51 | Formononetin | 0.71 | M+H | 269.0805 | -1.24917 | C16H12O4 |
| 52 | 5-methylcoumarin | 0.58 | M+NH4 | 178.0859 | -2.0186 | C10H8O2 |
